# Differential Expression of Long Noncoding RNAs between Sperm Samples from Diabetic and Non-Diabetic Mice

**DOI:** 10.1371/journal.pone.0154028

**Published:** 2016-04-27

**Authors:** Guang-Jian Jiang, Teng Zhang, Tian An, Dan-Dan Zhao, Xiu-Yan Yang, Dong-Wei Zhang, Yi Zhang, Qian-Qian Mu, Na Yu, Xue-Shan Ma, Si-Hua Gao

**Affiliations:** 1 Diabetes Research Center of Beijing University of Chinese Medicine, Beijing, China; 2 State Key Laboratory of Stem Cell and Reproductive Biology, Institute of Zoology, Chinese Academy of Sciences, Beijing, China; Qingdao Agricultural University, CHINA

## Abstract

To investigate the potential core reproduction-related genes associated with the development of diabetes, the expression profiles of long noncoding RNA (lncRNA) and messenger RNA (mRNA) in the sperm of diabetic mice were studied. We used microarray analysis to detect the expression of lncRNAs and coding transcripts in six diabetic and six normal sperm samples, and differentially expressed lncRNAs and mRNAs were identified through Volcano Plot filtering. The function of differentially expressed mRNA was determined by pathway and gene ontology (GO) analysis, and the function of lncRNAs was studied by subgroup analysis and their physical or functional relationships with corresponding mRNAs. A total of 7721 lncRNAs and 6097 mRNAs were found to be differentially expressed between the diabetic and normal sperm groups. The diabetic sperm exhibited aberrant expression profiles for lncRNAs and mRNAs, and GO and pathway analyses showed that the functions of differentially expressed mRNAs were closely related with many processes involved in the development of diabetes. Furthermore, potential core genes that might play important roles in the pathogenesis of diabetes-related low fertility were revealed by lncRNA- and mRNA-interaction studies, as well as coding-noncoding gene co-expression analysis based on the microarray expression profiles.

## Introduction

Type 2 diabetes mellitus (T2DM) has a variety of structural and functional effects on the male reproductive system [1]. T2DM could have detrimental effects on male sperm quality, including motility and DNA integrity. Epigenetic modifications are essential during spermatogenesis. Epigenetic regulation represents chromatin modifications, including DNA methylation, histone modifications, nucleosome remodeling, and noncoding RNAs (ncRNAs) [2–4]. Paternal T2DM may influence epigenetic modification during spermatogenesis, and this epigenetic dysregulation can be inherited transgenerationally through the mammalian germline, thereby increasing the risk of diabetes in offspring [5,6]. However, most mechanisms associated with T2DM-induced low fertility in males and inferior quality in offspring remain unknown. Increasing molecular evidence suggests that programming and inheritance of parental DNA methylomes in gametes play an important role in phenotype transmission from parents to offspring [7–10]. However, extensive studies suggest that ncRNAs can block or regulate DNA methylation [11,12].

It is estimated that protein-coding genes account for only ~5% to ~10% of the mammalian genome, meaning that a large number of transcripts do not encode proteins. Studies based on transcriptome profiles revealed potential core genes that result in long noncoding RNAs (lncRNAs), commonly defined as ncRNAs longer than 200 bp [13]. These genes were previously considered as ‘junk’ in the genome; however, emerging evidence indicated that lncRNAs preformed a wide variety of functions, including involvement in various pathophysiologic processes and human diseases [14–17]. Their corresponding functions associated with diabetic sperm remain relatively unexplored.

In this study, portions of lncRNAs adjacent or homologous to protein-coding genes were determined by searching the genome bioinformatics database. Microarray mRNA expression data were obtained for the sperm of mice. Our study provides insights into the functional interactions of lncRNA and mRNA, and offers new theories for the pathogenesis and treatment of diabetic reproductive dysfunction. Our results indicate that lncRNA may be a novel regulatory target associated with spermatogenesis in men with T2DM.

## Materials and Methods

### Ethics Statement

This study was approved by the Animal Care Committee of the Institute of Zoology, Chinese Academy of Sciences. All animal manipulations were undertaken according to the guidelines of the Animal Care Committee. For specific details and steps, see the following section.

### Sperm Collection

Sperm was collected from 22-week-old C57BL/6J and KK-Ay mice (Hua Fu Kang Company, Beijing, China). KK-Ay mice showed moderate hyperglycemia and obesity typical in the T2DM models, significantly improved glucose intolerance, and insulin resistance. Sperm from the epididymis were placed in preheated human tubal fluid culture and centrifuged at 1000x rpm for 5 min. Sperm capacitation was measured for 30 min, and then the sperm supernatant fluid was centrifuged again and collected.

### RNA Isolation and Quality Control

Total RNA was isolated with TRIzol reagent (Invitrogen, Carlsbad, CA, USA) and purified with an RNeasy Mini Kit (Qiagen, Hilden, Germany) according to manufacturer protocol. The NanoDrop ND-1000 was used to measure RNA quantity, and RNA quality was tested using the Agilent 2100 Bioanalyzer (Agilent Technology, Santa Clara, CA, USA). RNA integrity was assessed by standard denaturing agarose gel electrophoresis.

### RNA Labeling and Array Hybridization

Arraystar Mouse LncRNA Microarray version 3.0 was designed for the global profiling of mouse lncRNAs and protein-coding transcripts, with ~35,923 lncRNAs and 24,881 coding transcripts detected. Sample preparation and microarray hybridization were performed based on manufacturer standard protocols with minor modifications. An Arraystar RNA Flash Labeling Kit (Arraystar, Rockville, MD, USA) was used for sample labeling. Hybridization was performed in SureHyb Hybridization Chambers (Agilent Technology). After washing, the arrays were scanned using an Agilent DNA Microarray Scanner (Agilent Technology).

### Microarray Analysis

Agilent Feature Extraction software version 11.0.1.1 (Agilent Technology) was used to analyze the acquired array images. Quantile normalization and subsequent data processing were performed using the Agilent GeneSpring GX version 12.1 software package (Agilent Technology). After quantile normalization of the raw data, lncRNAs and mRNAs showing statistically significant differences in expression between the two groups were identified through *p*-value/FDR filtering. Differentially expressed lncRNAs and mRNAs between the two samples were identified through fold-change filtering. Pathway and gene ontology (GO) analyses were applied to determine the functional roles of the differentially expressed mRNAs. Hierarchical clustering and combined analysis were performed using in-house scripts.

### Quantitative Real-Time PCR

Real-time PCR was used to verify microarray results. Total RNA was extracted from six frozen samples of diabetic mouse sperm and six samples of control mouse sperm. Total RNA (2 μg) was converted to cDNA according to manufacturer protocol, and lncRNA and mRNA expression was measured by quantitative PCR using SYBR Premix ExTaq and an MX3000 instrument. The genes and primers used in this study are shown in S1 and S2 Tables. PCR was performed in a reaction that included 5 μL 2× PCR master mix, 0.5 μL forward primer (10 μM), 0.5 μL reverse primer (10 μM), 2 μL cDNA. The quantitative real-time PCR reaction was as follows: an initial denaturation step of 10 min at 95°C; 40 cycles of 95°C for 10 s, 60°C for 60 s, and 95°C for 15 s; and a final step of slow heating from 60°C to 99°C. All samples were normalized to GAPDH to calculate relative lncRNA and mRNA concentrations.

### GO and Pathway Analyses for Differentially Expressed mRNA

GO analysis (http://www.geneontology.org) allows functional association of differentially expressed mRNAs using three structured networks of defined terms that describe gene-product attributes. The *p*-value denotes the significance of GO-term enrichment in the differentially expressed mRNA list. The *p*-value cut-off was set at 0.05.

Differentially expressed mRNAs screened by Volcano Plot filtering were further investigated to determine the functions of the genes and the pathways with which they were associated using the latest Kyoto Encyclopedia of Genes and Genomes (http://www.genome.jp/kegg) database. The *p*-value denotes the significance of the pathway, and was set to a cut-off of 0.05.

### lncRNA Classification and Subgroup Analysis

This analysis was based on co-expression relationships between differentially expressed enhancer lncRNAs and nearby (distance < 300 kb) coding genes (fold change > 2; *p* < 0.05).

Natural antisense transcripts are a specific class of lncRNAs that are derived from the reverse-strand and overlap-coding transcripts. Further analysis filtered differentially expressed natural antisense transcripts, including lncRNAs, and their nearby (distance < 300 kb) coding genes (fold change > 2; *p* < 0.05).

According to the noncoding list, the weighted co-expression network was constructed by calculating a pairwise correlation matrix between all probe sets across microarray samples. The resulting Pearson correlation matrix was transformed into an adjacency matrix. We weighed the Pearson correlation results by taking their absolute value and raising them to the power. The nodes of the co-expression network correspond to gene expression, and edges between genes are determined by the correlations. Pearson correlation coefficients (PCCs) were calculated between the coding and noncoding results with the use of R statistical analysis (PCC ≥ 0.999; https://www.r-project.org/). The coding-noncoding (CNC) network was constructed using Cytoscape v2.8.2 software (http://www.cytoscape.org/).

## Results

### Sperm from Diabetic Mice Demonstrate Altered lncRNA and mRNA Expression Patterns

**Fig 1:**
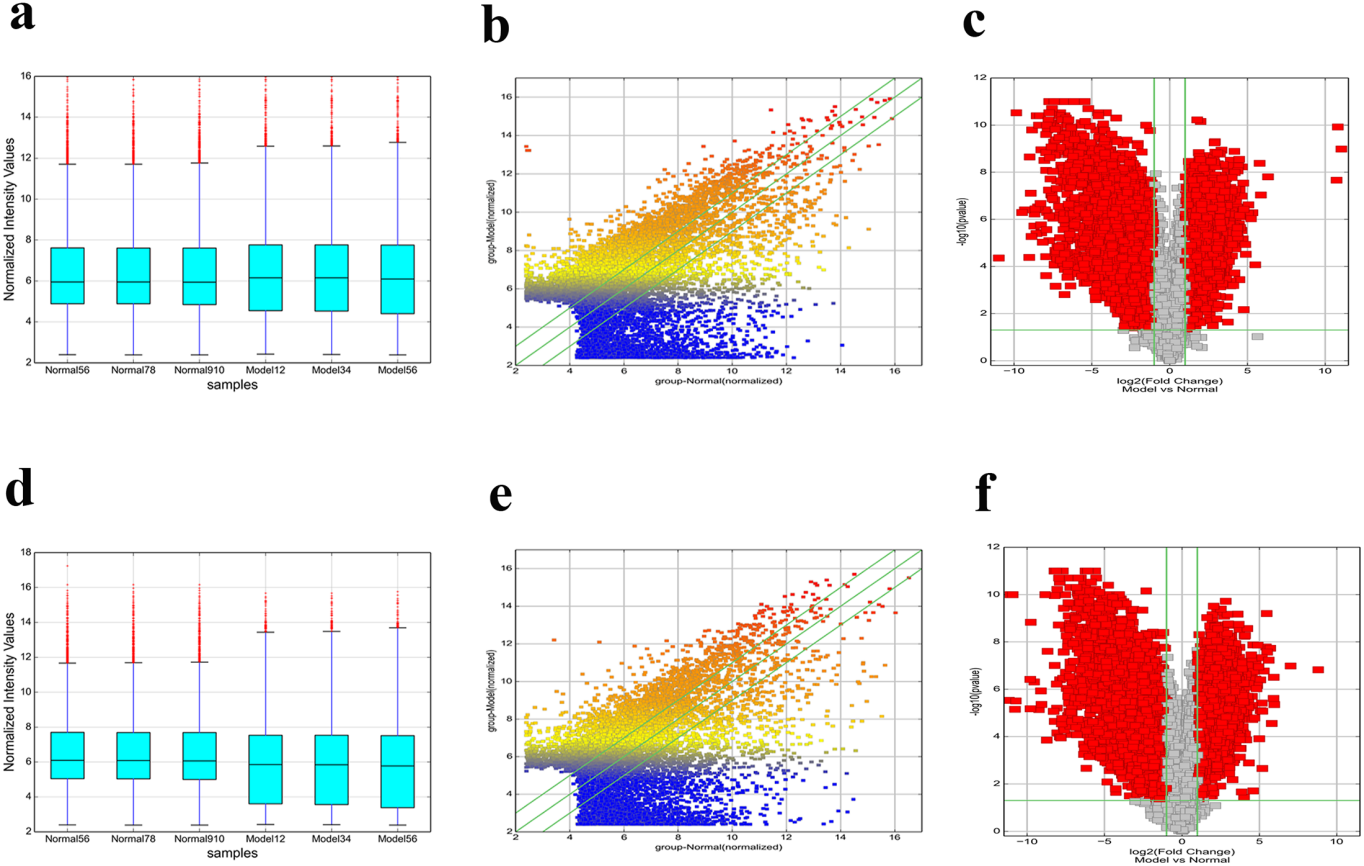
Plots of lncRNA and mRNA. Box plot of (A) lncRNA and (D) mRNA. Scatter plot of (B) lncRNA and (E) mRNA. Volcano plot of (C) lncRNA and (F) mRNA. Plots were constructed using fold-change and p values, enabling visualization of the relationship between fold change (magnitude of change) and statistical significance (which takes both magnitude of change and variability into consideration). The vertical lines correspond to a 2-fold change in expression (up or down), and the horizontal line represents *p* = 0.05. The red point in the plot represents the differentially expressed genes with statistical significance.

**Fig 2:**
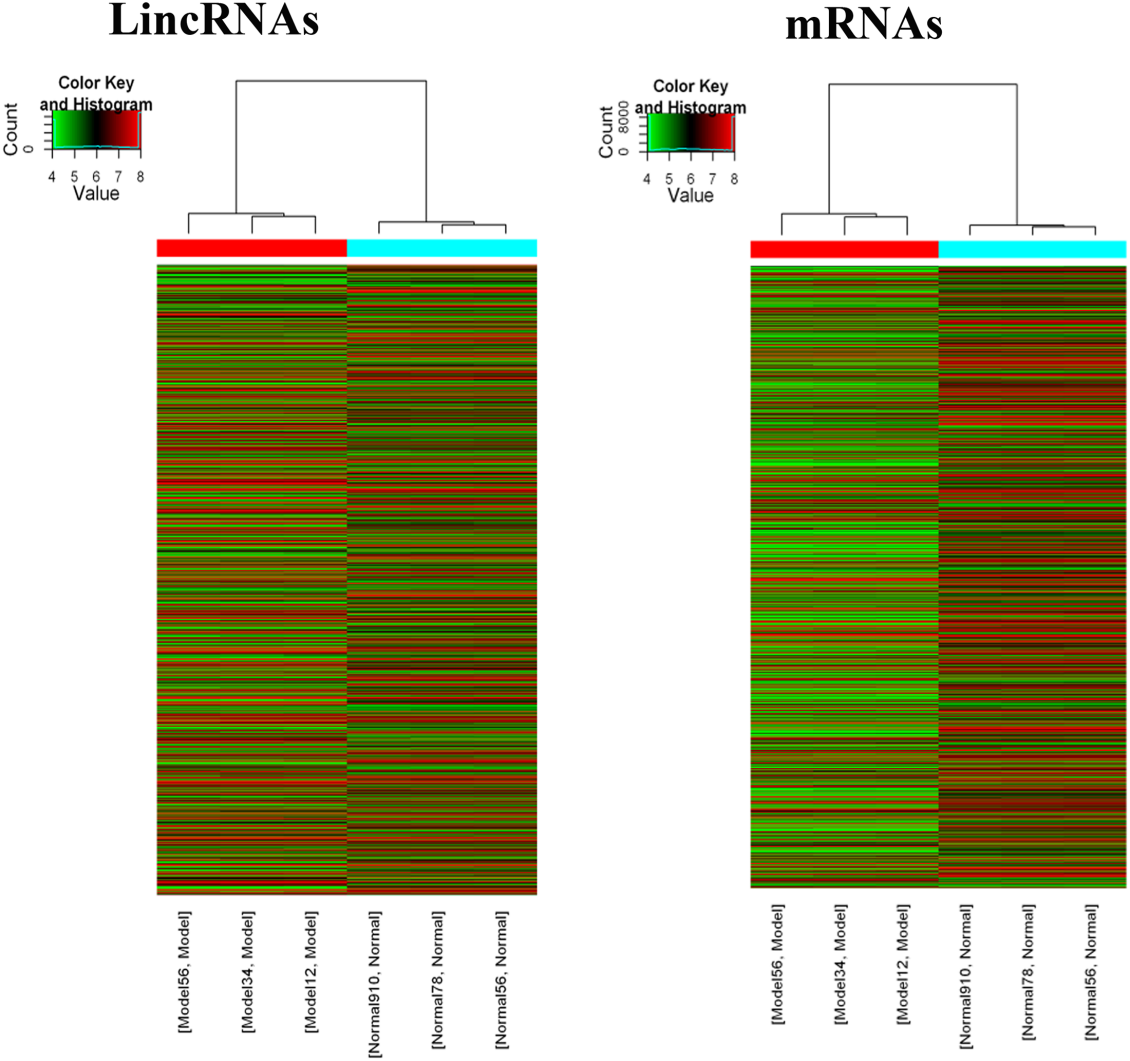
Hierarchical clustering of lncRNA and mRNA by Z-score. Based on the expression levels of (A) lncRNAs or (B) mRNAs, the six samples were classified into two groups (sperm from diabetic or control mice). The dendrogram shows the relationships among the expression levels of samples. Red indicates high relative expression, and green indicates low relative expression.

**Fig 3:**
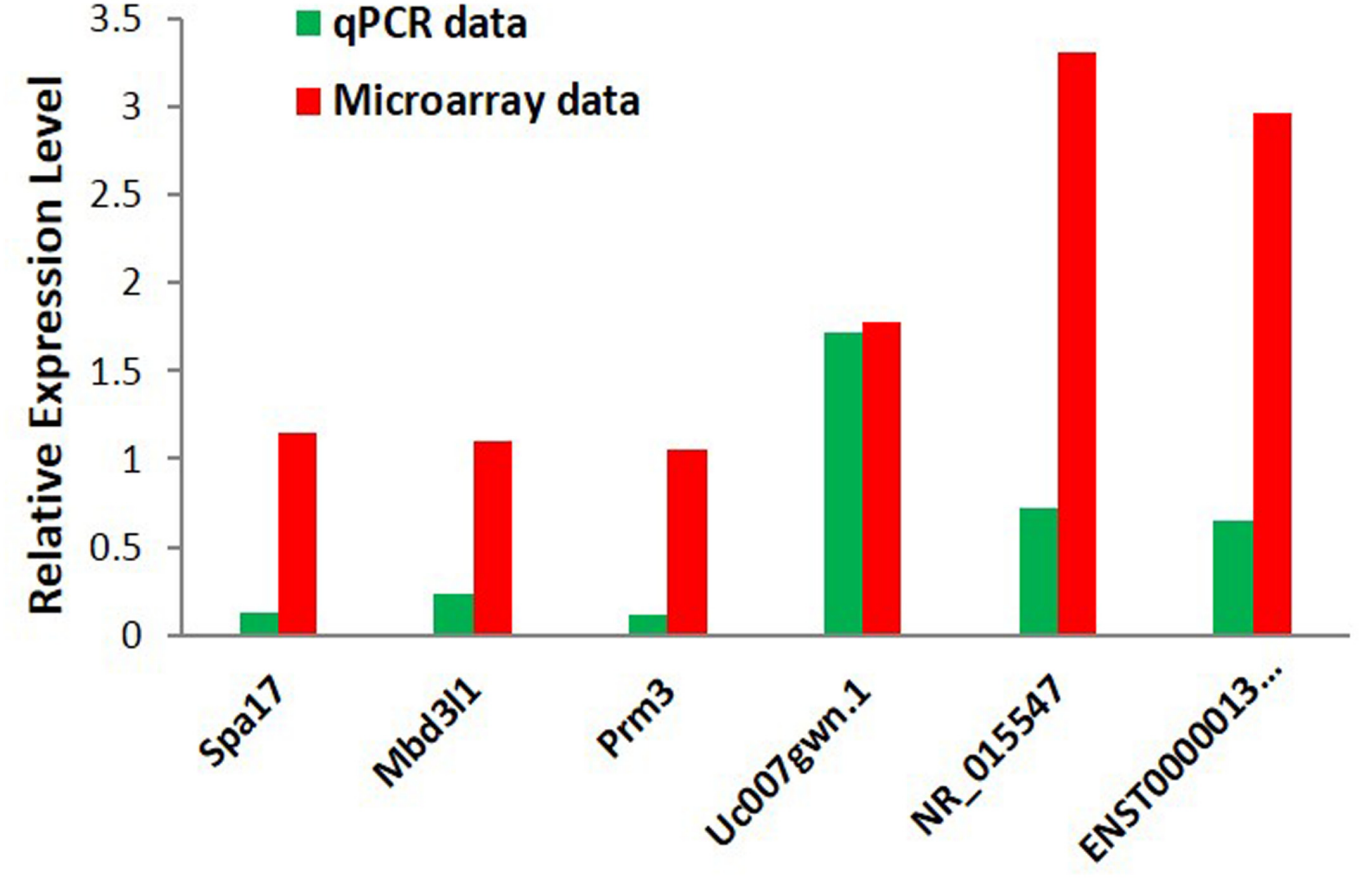
Microarray and quantitative PCR. Microarray and quantitative PCR for lncRNAs (Uc007gwn.1, NR_015547, ENSMUST00000134455) and mRNAs (Spa17, Mbd311, prm3). The quantitative PCR results were consistent with the microarray data.

The lncRNA and mRNA expression profiles were detected from six microarray experiments, indicating that 4134 upregulated and 3407 downregulated lncRNAs, 2590 upregulated and 3507 downregulated coding transcripts, from diabetic and control mice sperm samples respectively. (GEO dataset: GSE51146; Fig 1; S5 and S6 Tables). Hierarchical clustering analysis revealed that the expression of these genes enabled the samples to be readily classified into two groups, i.e., the diabetic and control groups (Fig 2). The expression levels of three selected lncRNAs (Uc007gwn.1F, NR_015547, and ENSMUST00000134455) and three mRNAs (NM_013638Prm3, NM_028557Mbd3l1, and NM_011449Spa17), as determined by quantitative real-time PCR, were consistent with the microarray results (Fig 3), verifying the accuracy and reliability of the microarray data.

### Enrichment Analysis of Differentially Expressed Genes

**Fig 4:**
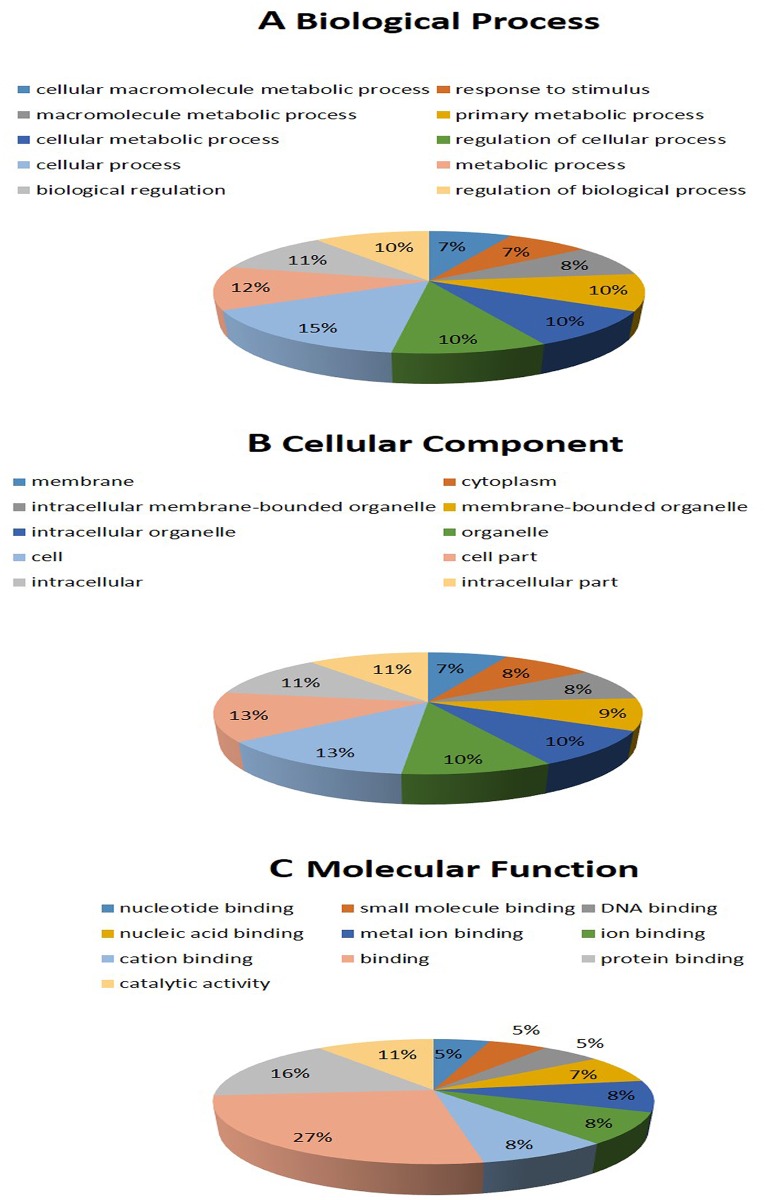
Gene ontology enrichment for lncRNA targets in the category of biological processes from sperm samples. Gene ontology analysis of lncRNA-target genes according to (A) biological process, (B) cell component, and (C) molecular function.

GO analysis revealed the functions of differentially expressed (both upregulated and downregulated) mRNA in sperm samples extracted from diabetic mice (Fig 4). The functions of these mRNAs are related with many processes that are important in diabetic reproductive pathogenesis, such as RNA processing, developmental processes, spermatogenesis, mRNA processing, and male gamete generation, as well as molecular functions, including insulin-like growth factor receptor binding, MAP kinase phosphatase activity, nucleotide binding, and cellular functions, such as voltage-gated calcium channel complexes and mitochondrial respiratory chain complex I.

Pathway analysis indicated that the upregulated mRNAs in the diabetic samples participated in DNA replication and cell signaling (the Wnt signaling pathway, transcriptional misregulation in cancer, and the Hippo signaling pathway; S7 Table). However, the downregulated mRNAs were involved in the PPAR signaling pathway, mRNA surveillance pathway, RNA degradation, protein processing in the endoplasmic reticulum, and processes related to many diseases (Huntington's disease, Parkinson's disease, and Alzheimer's disease) associated with aging (S8 Table).

### Subgroup Analysis Revealed Potential Diabetes-Related lncRNAs and mRNAs

A total of 25 differentially expressed lncRNAs with enhancer-like functions between the diabetic and control groups were filtered (fold change > 2; *p* < 0.05), with the 10 most significantly upregulated and downregulated enhancer lncRNAs are listed in S3 Table. A total of 217 differentially expressed lncRNAs with natural antisense functions between the diabetic and control groups were filtered (fold change > 2; *p* < 0.05), with the 20 most significantly upregulated and downregulated antisense lncRNAs listed in S4 Table.

**Fig 5:**
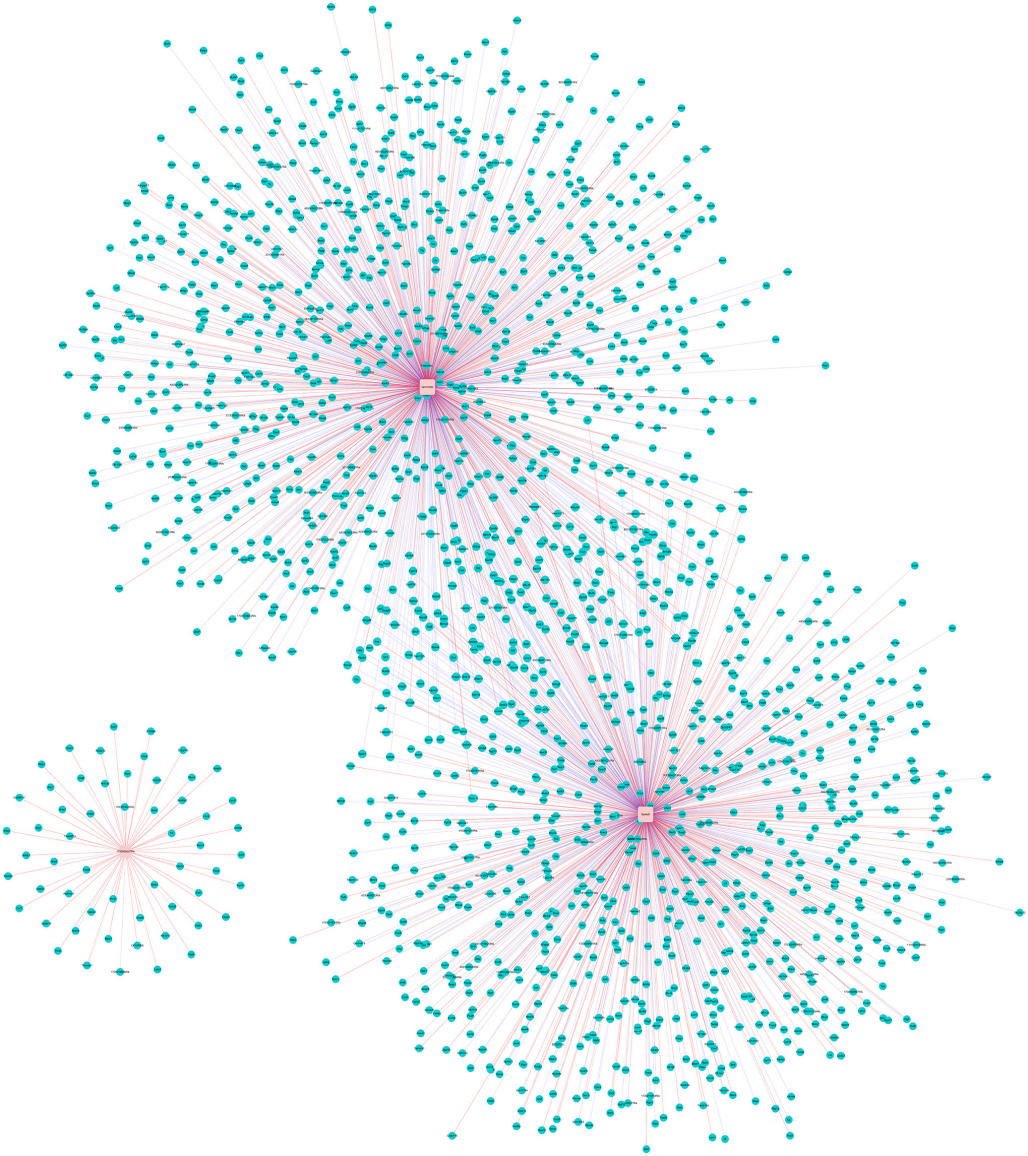
The color-coded CNC network (1700009J07Rik, Spats21, and Gm16180). Blue (RGB: 0, 205, 205) are round nodes that represent coding genes, and pink (RGB: 255, 204, 204) are square nodes representing noncoding genes. The red solid line between the two nodes represents a positive correlation, and the blue dashed line represents a negative correlation. The difference between nodes associated with upregulated genes is marked with red, while the difference between nodes associated with downregulated genes is marked with green.

The exact functions of the majority of such transcripts are still unknown. Here, we reported three computational annotations of lncRNA functions based on public microarray expression profiles. A CNC gene co-expression network was constructed from re-annotated Affymetrix Mouse Genome Array data (Fig 5).

## Discussion

Recent advances in microarray technology have enabled global analyses of spermatozoal lncRNAs and mRNAs, which have contributed to the understanding of the roles played by lncRNA in sperm from diabetic patients and the discovery of clinical markers for diabetic male infertility. In this study, we have built complete lncRNA and mRNA expression profiles of sperm derived from diabetic and control mice using gene expression microarrays. Spermatozoal lncRNA profiling has the potential to identify sperm-associated factors related to T2DM.

A total of 7721 lncRNAs (4314 upregulated and 3407 downregulated) and 6097 mRNAs (2590 upregulated and 3507 downregulated) were found to be differentially expressed between sperm samples from the diabetic and control groups.

Enhancers are important elements that regulate gene expression, with many possibly participating in pathological mechanisms associated with disease. Therefore, we analyzed our microarray data to screen differentially expressed lncRNAs with enhancer-like functions between the diabetic and control samples. The targets with the largest fold changes and the smallest *p* values were deemed possible candidates for further study.

Regulating the expression of adjacent genes is one mechanism associated with lncRNAs, although distant targets might also be regulated under certain conditions. We performed co-expression analysis of mRNA transcripts and from genes located adjacent (<300 kb) to enhancer-like lncRNA. When the expression profiles associated with both the mRNA and the lncRNA transcripts changed significantly between sperm samples from diabetic and control mice, this was taken to indicate the existence of interactions between the lncRNA and the gene associated with the mRNA transcript. We have identified some potential lncRNAs, such as Rpl31-ps7, AB352974, Rpl35a-ps7, Rn4.5s, and XLOC_01985, associated with diabetes-related phenotypes.

Sperm formation requires tightly regulated gene expression and unique chromatin remodeling. Here, we found that the expression of the lysine-specific histone H3 methyltransferase (Ezh2) was upregulated by three lncRNA enhancers (Rpl31-ps7, AB352974, and Rpl35a-ps7), which were downregulated, and two lncRNA enhancers (Rn4.5s and XLOC_01985), which were upregulated in sperm samples from diabetic mice. Molecular analyses established that the polycomb-group protein Ezh2, which is involved in maintaining the transcriptional repressive state of genes over successive cell generations, is a key effector of the nuclear apical region of round spermatids (specialized epigenetic regions), where methylation of histones serves a role in spermiogenic chromatin remodeling [18]. Further research is required to understand the exact mechanism involving Ezh2 association with T2DM sperm epigenetics.

In order to determine additional lncRNA functions, we focused on antisense lncRNAs. Natural antisense lncRNAs are RNA molecules that are transcribed from the antisense strand and partially overlap with well-defined spliced- or intronless-sense RNAs. Antisense lncRNAs were initially considered to be transcriptional noise; however, there is now considerable evidence showing that antisense lncRNA can regulate sense mRNA at the transcriptional and post-transcriptional levels through a variety of mechanisms [19,20]. Several natural antisense transcripts have been described in mature spermatozoa [21–23], and spermatogenesis is partially regulated through the action of lncRNAs, some of which are likely antisense [24,25]. The function of some natural antisense lncRNAs in sperm maturation, fertilization, and early embryo development remains to be explored. Here, we discovered some natural antisense lncRNAs possibly involved in regulating expression of neighboring genes or more distant genes through various mechanisms. Specifically, Hnrnpab, which was downregulated by lncRNA AK144334 in the sperm sample from diabetic mice, plays an important role in spermatogenesis by regulating stage-specific translation of testicular mRNAs [26]. We anticipate that additional natural antisense lncRNAs will be found to be potential regulators of diabetic male fertility.

The functions of most lncRNAs have not been determined, and there is no existing database available housing their functional annotations. We attempted to correlate mRNA and lncRNA expression in order to indirectly determine lncRNA functions based on the study of its corresponding mRNA. We systematically analyzed the functions of differentially expressed mRNAs by GO annotation and pathway analysis. We found that the functions associated with these targets were related to many aspects of diabetic progression and were involved in many spermatogenesis-associated pathways, such as glucose and lipid metabolism and oxidative phosphorylation. Recent investigations highlighted the role of the Wnt signaling pathway in metabolic homeostasis and its implications in diabetes, as well as other metabolic diseases. It was confirmed that high glucose can activate this pathway [27], and many researchers have focused on the importance of its role during embryonic development. Wnt signaling also plays an important role in regulating mammalian spermatogenesis [28], with studies indicating that it is essential for adult spermatogenesis, which supports the growing belief that its disruption may underpin certain cases of male infertility [29]. Additionally, the Hippo signaling pathway plays a major role in organ-growth control, and Park et al established the transcriptional co-activators YAP and TAZ as critical mediators of alternative Wnt signaling [30]. Interestingly, transcripts associated with both the Wnt and Hippo pathways were upregulated in the sperm from diabetic mice, indicating that the regulatory mechanisms and biological functions involved in the Hippo and Wnt pathways might reveal potential targets of therapeutic intervention for diabetic male infertility.

Spermatogenesis is a complex and highly regulated biological process. The current understanding of lncRNAs role in regulating spermatogenesis remains incomplete. Our microarray data provides additional evidence for the prediction and annotation of lncRNA involvement in diabetic male fertility. We anticipate that lncRNAs will become attractive biomarkers to enhance the investigative and diagnostic potential associated with research into male infertility.

## Supporting Information

S1 Table LincRNA primers used in quantitative real-time PCR. (DOC)

S2 Table The mRNA primers used in quantitative real-time PCR. (DOC)

S3 Table Enhancer LncRNAs nearby coding gene. (DOC)

S4 Table Natural antisense LncRNAs nearby coding gene. (DOC)

S5 Table Upregulated and downregulated lncRNAs from diabetic and control mice sperm samples. (XLS)

S6 Table Upregulated and downregulated coding transcripts from diabetic and control mice sperm samples. (XLS)

S7 Table The upregulated mRNA involved in pathways in the diabetic samples. (XLS)

S8 Table The downregulated mRNA involved in pathways in the diabetic samples. (XLS)
