# Supplementary material for: Differential Expression of Long Noncoding RNAs between Sperm Samples from Diabetic and Non-Diabetic Mice
Source: PLoS One. 2016 Apr 27;11(4):e0154028. doi: 10.1371/journal.pone.0154028 (PMC4847876; doi:10.1371/journal.pone.0154028)
Supplement: S1 Table — (DOC) [file pone.0154028.s001.doc]

**Supplementary Tables 1.** LincRNA primers used in quantitative real-time PCR

| **Primers Seqname Sequences** |
| --- |
| GAPDH(MOUSE)F 5’GTTGTCTCCTGCGACTTCA3’  GAPDH(MOUSE)R 5’GCCCCTCCTGTTATTATGG3’ |
| Uc007gwn.1F 5’CAATGGCAGCACCTAAAATAAG3  Uc007gwn.1R 5’TTGGCAGAACAGGAGAAGTG3’  NR_015547F 5’ATCTCACCCCTCATTCCAAG3’ NR_015547R 5’AACTGGAGCAGGTTGAGAAGAG3’  ENSMUST00000134455F 5’CCTCTACTTACTGCCCACACC3’  ENSMUST00000134455R 5’TCTTATCCATCATTCCCTTCC3’ |
