# Supplementary material for: Differential Expression of Long Noncoding RNAs between Sperm Samples from Diabetic and Non-Diabetic Mice
Source: PLoS One. 2016 Apr 27;11(4):e0154028. doi: 10.1371/journal.pone.0154028 (PMC4847876; doi:10.1371/journal.pone.0154028)
Supplement: S2 Table — (DOC) [file pone.0154028.s002.doc]

**Supplementary Tables 2.** The mRNA primers used in quantitative real-time PCR

| **Primers Seqname Sequences** |
| --- |
| GAPDH(MOUSE)F 5’GTTGTCTCCTGCGACTTCA3’  GAPDH(MOUSE)R 5’GCCCCTCCTGTTATTATGG3’ |
| NM_013638Prm3F 5’GAGCCCGAAAAGCAAGAGA3’  NM_013638Prm3R 5’TTCTGAATGTCCTCTGGCGT3’  NM_028557Mbd3l1 F 5’CACATCCGTACCCACCCTT3’  NM_028557Mbd3l1R 5’GTTTGCCTTCCTTGACCCC3’  NM_011449Spa17F 5’CGGTTACCCAGCAACGAGA3’  NM_011449Spa17R 5’AATCTCCCGTGTCAGCCCT3’ |
