# Supplementary material for: Differential Expression of Long Noncoding RNAs between Sperm Samples from Diabetic and Non-Diabetic Mice
Source: PLoS One. 2016 Apr 27;11(4):e0154028. doi: 10.1371/journal.pone.0154028 (PMC4847876; doi:10.1371/journal.pone.0154028)
Supplement: S3 Table — (DOC) [file pone.0154028.s003.doc]

**Supplementary Tables 3.** Enhancer LncRNAs nearby coding gene

| **GeneSymbol** | **P-value- LncRNAsRNAs** | **Fold change- LncRNAs- LncRNAs** | **Regulation - LncRNAs LncRNAs** | **Nearby**  **GeneSymbol Symbol** | **P value**  **- mRNAsNAs** | **Fold change mRNAs - mRNAs** | **Regulation - mRNAs mRNAs** |
| --- | --- | --- | --- | --- | --- | --- | --- |
| Rpl31-ps7 | 0.0000 | 182.656 | down | Ezh2 | 0.0000 | 3.563 | up |
| 1110038B12Rik | 0.0000 | 17.975 | down | Nfkbil1 | 0.0004 | 13.773 | down |
| Gm4883 | 0.0000 | 218.059 | down | Psenen | 0.0000 | 5.131 | down |
| Gm4883 | 0.0000 | 218.059 | down | Nfkbid | 0.0003 | 2.568 | up |
| 1110038B12Rik | 0.0000 | 18.091 | down | Nfkbil1 | 0.0004 | 13.773 | down |
| AB352974 | 0.0000 | 8.344 | down | Ezh2 | 0.0000 | 3.563 | up |
| Gm11634 | 0.0000 | 20.327 | down | Etv4 | 0.0000 | 5.697 | up |
| AK141527 | 0.0000 | 10.317 | down | Mien1 | 0.0000 | 2.833 | down |
| Rpl35a-ps7 | 0.0000 | 161.119 | down | Ezh2 | 0.0000 | 3.563 | up |
| Pmis2 | 0.0002 | 17.012 | down | Psenen | 0.0000 | 5.131 | down |
| Pmis2 | 0.0002 | 17.012 | down | Nfkbid | 0.0003 | 2.568 | up |
| Aldoart2 | 0.0008 | 12.396 | down | Nfkbia | 0.0000 | 26.935 | down |
| Gm20419 | 0.0038 | 5.453 | down | Nfkbil1 | 0.0004 | 13.773 | down |
| AK201601 | 0.0000 | 9.171 | up | Aes | 0.0000 | 4.840 | down |
| AK201601 | 0.0000 | 9.171 | up | Aes | 0.0002 | 2.566 | down |
| A530013C23Rik | 0.0000 | 7.086 | up | Cebpb | 0.0000 | 13.009 | down |
| TCONS_00033998 | 0.0000 | 5.602 | up | Tle3 | 0.0000 | 3.650 | up |
| TCONS_00033998 | 0.0000 | 5.602 | up | Tle3 | 0.0000 | 2.147 | up |
| TCONS_00033998 | 0.0000 | 5.602 | up | Tle3 | 0.0000 | 4.422 | up |
| 1110038B12Rik | 0.0000 | 4.322 | up | Nfkbil1 | 0.0004 | 13.773 | down |
| Rn4.5s | 0.0000 | 2.556 | up | Ezh2 | 0.0000 | 3.563 | up |
| XLOC_019521 | 0.0000 | 16.185 | up | Pcolce | 0.0000 | 4.685 | up |
| XLOC_019850 | 0.0000 | 2.025 | up | Ezh2 | 0.0000 | 3.563 | up |
| Gm13376 | 0.0009 | 6.232 | up | Etl4 | 0.0000 | 6.382 | up |
| Gm13376 | 0.0009 | 6.232 | up | Etl4 | 0.0000 | 4.208 | up |
