# Supplementary material for: Differential Expression of Long Noncoding RNAs between Sperm Samples from Diabetic and Non-Diabetic Mice
Source: PLoS One. 2016 Apr 27;11(4):e0154028. doi: 10.1371/journal.pone.0154028 (PMC4847876; doi:10.1371/journal.pone.0154028)
Supplement: S4 Table — (DOC) [file pone.0154028.s004.doc]

**Supplementary Tables 4.** Natural antisense LncRNAs nearby coding gene

| **GeneSymbol** | **P-value - LncRNAs** | **Fold change - LncRNAs** | **Regulation - LncRNAs** | **Nearby GeneSymbol** | **P value - mRNAs** | **Fold change - mRNAs** | **Regulation - mRNAs** |
| --- | --- | --- | --- | --- | --- | --- | --- |
| AK133391 | 0.000 | 113.542 | down | Grin2d | 0.000 | 4.347 | up |
| AK035123 | 0.000 | 107.366 | down | Recql | 0.000 | 3.976 | up |
| AK035123 | 0.000 | 107.366 | down | Recql | 0.000 | 5.629 | down |
| AK035123 | 0.000 | 107.366 | down | Recql | 0.000 | 6.302 | up |
| Kcnmb4os1 | 0.000 | 60.540 | down | Kcnmb4 | 0.000 | 13.435 | down |
| Slfn5os | 0.000 | 50.265 | down | Unc45b | 0.000 | 2.528 | up |
| AK131746 | 0.000 | 39.755 | down | Itpr3 | 0.000 | 2.160 | up |
| Gm16104 | 0.000 | 39.365 | down | Nfic | 0.000 | 5.077 | up |
| Nrd1 | 0.000 | 35.185 | down | Osbpl9 | 0.000 | 8.091 | down |
| Nrd1 | 0.000 | 35.185 | down | Osbpl9 | 0.000 | 5.069 | up |
| Gm11149 | 0.000 | 31.720 | down | Ncam1 | 0.000 | 6.919 | up |
| AK005812 | 0.000 | 30.563 | down | Rgcc | 0.000 | 4.207 | down |
| 1700012B15Rik | 0.000 | 22.763 | down | Rab10 | 0.000 | 5.927 | down |
| Fam166a | 0.000 | 22.646 | down | Tubb4b | 0.000 | 5.998 | up |
| A430018G15Rik | 0.000 | 21.487 | down | Cacnb4 | 0.000 | 3.988 | up |
| Gm15415 | 0.000 | 20.761 | down | Tenm4 | 0.000 | 13.075 | up |
| AK144334 | 0.000 | 19.678 | down | Hnrnpab | 0.000 | 5.026 | down |
| AK144334 | 0.000 | 19.678 | down | Hnrnpab | 0.000 | 10.614 | down |
| AK157091 | 0.000 | 18.676 | up | N4bp2l2 | 0.000 | 25.587 | down |
| 4930486I03Rik | 0.000 | 17.034 | down | Pkhd1 | 0.000 | 4.277 | up |
|  |  |  |  |  |  |  |  |
|  |  |  |  |  |  |  |  |
